# The pharmacological protection of renal function in patients undergoing cardiac surgery

**DOI:** 10.12669/pjms.315.7679

**Published:** 2015

**Authors:** Emine Bilge Narin, Ilhan Oztekin, SeherDeniz Oztekin, Betul Ogutmen

**Affiliations:** 1 Emine Bilge Narin, Anesthesiologist, Department of Cardiac Surgery Intensive Care, Medicana Hospital, Umraniye, Istanbul, Turkey; 2 Ilhan Oztekin, Professor, Department of Anesthesiology & Reanimation, Faculty of Medicine, Trakya University, Edirne, Turkey; 3 Seher Deniz Oztekin, PhD. Professor in Nursing, Department of Surgical Nursing, Florence Nightingale Faculty of Nursing, Istanbul University, Istanbul, Turkey; 4 Betul Ogutmen, Associate Professor, Department of Nephrology, DrSiyamiErsek Research and Training Hospital for Cardiovascular Surgery, Istanbul, Turkey

**Keywords:** Coronary artery surgery, cystatin-c, Dopamine, mannitol, microalbumin, urine microalbumin/creatinine ratio

## Abstract

**Objectives:**

To compare the effects of different routes and timings of administration of dopamine and mannitol used to alleviate the adverse effects of prolonged cardiopulmonary bypass (CPB) on renal functions in coronary artery surgery.

**Methods:**

Group I (n: 25 patients): Mannitol 1 g/kg was added into the priming solution for CPB. Group II (n: 25 patients): IV dopamine was administered at a dose of 2 μg/kg/min during the time period between anesthesia induction and end of surgery. Group III (n: 25 patients): IV dopamine was administered at a dose of 2 μg/kg/min during the time period between anesthesia induction and end of surgery and mannitol 1 g/kg was added into the priming solution for CPB. Group IV (n: 25 patients) (Controls): Furosemide was given when the urine output was low.

**Results:**

There was a significant increase in post operative urine microalbumin/creatinine ratio in all groups (p < 0.05), even increase of cystatin-c in Groups I, II and III (p < 0.01).

**Conclusions:**

We believe that concurrent use of dopamine infusion (2 μg/kg/min) with mannitol (1 g/kg) during CPB may represent a more effective strategy for the prevention of the untoward effects of CPB on renal functions.

## INTRODUCTION

In around 30-40% of the patients undergoing coronary artery bypass grafting (CABG) surgery renal functions are adversely affected, with 1 to 3% developing acute renal failure (AFR).^1^ A number of different etiological factors are responsible for the reduction in renal perfusion and for the ischemic injury. For instance, during cardiopulmonary bypass (CPB) an increasing level of renal vascular resistance is accompanied by a 30% reduction in renal blood flow. The high morbidity and mortality rates in ARF are seen, even in the presence of supportive treatment with hemodialysis and high dose inotropic support.^1^,^2^

Low dose dopamine (2-4 μg/kg/min) exerts its actions through its effects on the dopaminergic receptors in the renal artery (i.e. DA1 and DA2). Dopamine also increases the cardiac output, maintains renal perfusion, decreases renal metabolism, and results in diuresis.^3^

Mannitol administration during CPB allows maintenance of the glomerular capillary pressure by preventing tubular obstruction. It also reduces the plasma levels of hydrogen peroxide free oxygen radicals, lowers the ischemia-associated protein leakage from renal vasculature, and prevents renal injury.^1^,^4^

By examining several renal function parameters, this study aimed to compare the effects of different routes and timings of dopamine and mannitol administration, which are used to alleviate the adverse effects of prolonged CPB and to preserve renal function.

## METHODS

A total of 100 ASA III patients with normal renal function, EF of greater than 40%, normal protein and electrolyte levels who were scheduled for elective CABG surgery at the Dr Siyami Ersek Research and Training Hospital for Cardiovascular Surgery were included in this study. The study protocol was approved by ethics committee of the hospital. Also, the informed consent was obtained from each patient.

Exclusion criteria included the use of ACE (angiotensin converting enzyme) inhibitors or diuretics, recent myocardial infarction, anemia, diabetes, and use of radiological contrast medium within the last three days. Preoperative (preop) and postoperative Day 2 (postop) urinary microalbumin, urinary creatinine and serum cystatin-c values were compared.

For anesthesia induction, 2 mg/kg of propofol (Diprivan, AstraZeneca), 15 µg/kg/min of fentanyl (Fentanyl, Janssen-Cilag) and bolus intravenous (IV) 0.1 mg/kg of pancuronium (Pavulon, Schering-Plough) were given during 100% oxygen inhalation. Urine output was monitored through a urinary bladder catheter placed after intubation (Bicakcilar Urimeter 500, sterile closed urine measurement system=urofix) and a urinary flow measurement device was used for measuring hourly urine output. For anesthesia maintenance, 8 µg/kg/h of fentanyl and 2 mg/kg/h of propofol infusions as well as isoflurane (Forane likit, Abbott, 0.4 to 1.0%) by inhalation were given. Patients were assigned into the following two groups according to the method of dopamine and mannitol administration:

### Group I (n:25 patients)

Mannitol 1 g/kg, was added into the priming solution for CPB. Group II (n:25 patients): No mannitol was added to the priming solution. After the induction of anesthesia, IV dopamine was administered until the termination of surgery at a rate of 2 μg/kg/min. Group III (n:25 patients): Following the induction of anesthesia, IV dopamine was given at a rate of 2 μg/kg/min until the termination of surgery. Mannitol 1 g/kg was added into the priming solution. Group IV (n:25 patients) (Controls): No dopamine or mannitol was added into the priming solution and furosemide was given in patients with low urine output.

Except during CPB, a mean arterial pressure (MAP) of 65 to 100 mmHg and a heart rate (HR) of 60 to 100 bmp was targeted. Quick volume replacement was performed in patients experiencing hypotension. When this failed to restore target blood pressure levels, ephedrine was given. 1-3 µg/kg/min nitroglycerine was used for hypertensive episodes. Standard cardiac surgery cannulation was performed, and moderate hypothermia (esophageal temperature of 28 C) and blood cardioplegia were used for cardiac protection. A Biomedikus pump system (Minneapolis-Minnesota) and membrane oxygenator (Dideco 0.708 Simplex) were utilized for CPB.

The pumping priming solution consisted of lactated Ringer’s (30 ml/kg) + Heparin (1 mg/kg) + Mannitol 20% (1 g/kg) in Groups 1 and 3 in addition to 60 ml of NaHCO3. During CPB, the hematocrit value was kept between 20 and 25% (i.e. hemodilution). For systemic heparinization, heparin at a dose of 3-4 mg/kg was administered according to the initial activated coagulation time (ACT), which was maintained above 400 sec with 30 min ACT monitoring (Actalyke) intervals. For hemodynamic stabilization at the intensive care unit, a MAP of 50 to 100 mmHg was targeted. Blood transfusions were given to maintain the hematocrit level above 25%.

### Statistical analyses

SPSS (Statistical Package for Social Sciences) for Windows version 15.0 was used for statistical analyses. Oneway Anova test was used as a parametric test for between-group comparisons and Tukey HDS test was used for the determination of the group responsible for the difference, in addition to descriptive statistical methods (mean, standard deviation). For between-group comparisons, Kruskal Wallis test was used as a non-parametric test. Paired sample t test was used as a parametric test for within group comparisons, whereas Wilcoxon sign test was used as a non-parametric test. Chi-square test was used for the comparison of qualitative data. The results were evaluated at a 95% confidence interval and a p level of less than 0.05 considered an indication for statistical significance.

## RESULTS

Demographic characteristics of the patients are summarized in Table-I. There were no significant differences between the groups in terms of demographic characteristics. Despite a postoperative reduction in urinary creatinine in all groups, the reduction reached statistical significance in Group II (p < 0.05) (Table-II).

**Table-I:** Demographic characteristics of the groups.

	Group I	Group II	Group III	Group IV	♦p
	Mean±SD	Mean±SD	Mean±SD	Mean±SD	
Age	57.36±11.11	57.56±8.39	56.64±10.18	59.56±8.00	0.730
Height (cm)	165±4.1	169±8.6	67±5.2	168±6.1	0.880
Body weight (kg)	77±6.9	80±10	78±7.3	76±4.5	0.780
Duration of operation (min)	247±41.5	244±38.7	248±52.3	245±48.5	0.820
Cross-clamping time (min)	72±20.1	63±20.8	78±16.4	74±27.8	0.860
CPB time (min)	94±22.7	92±27.4	93±24.7	95±21.3	0.920
Gender n (%)					
Male	19 (76.0%)	20 (80.0%)	21 (84.0%)	19(76.0%)	♦p 0.919
Female	6 (24.0%)	5 (20.0%)	4 (16.0%)	6(24.0%)	

♦ : student t test; ♦ : Chi square test; Mean±SD: Mean±Standard deviation; CPB: Cardiopulmonary bypass; p<0.05.

**Table-II:** Between group and within group comparisons of the mean values of biochemical parameters indicative of acute renal failure.

		Group I	Group II	Group III	Group IV	♦p
		Mean±SD (Median)	Mean±SD (Median)	Mean±SD (Median)	Mean±SD (Median)
Urine creatinine (mg/dL)	Preop	133.81±76.40	163.22±81.34	154.12±73.05	149.16±100.45	0.654
	Postop Day 2	124.96±80.74	117.23±78.21	123.25±150.84	123.80±77.11	0.993
	♦p	0.680	0.039*	0.383	0.208	
Cystatin-C (mg/dL)	Preop	0.78±0.16	0.79±0.13	0.76±0.12	0.86±0.14	0.082
	Postop Day 2	0.93±0.20	0.93±0.22	0.85±0.12	0.92±0.22	0.396
	♦p	0.004**	0.001**	0.001**	0.167	
Urıne Microalbumin (mg/dL)	Preop	2.79±4.76 (1.15)	5.45±13.7 (2.20)	2.61±5.22 (0.78)	2.98±7.54 (1.00)	0.335
	Postop Day 2	9.29±18.44 (4.66)	5.67±6.33 (3.00)	2.73±2.19 (2.30)	5.35±7.70 (3.60)	0.688
	♦p	0.002**	0.031*	0.264	0.005**	
Urine microalbumin/creatinin ratio (mg/dL)	Preop	0.01±0.02 (0.012)	0.03±0.11 (0.008)	0.01±0.02 (0.005)	0.05±0.23 (0.008)	0.242
	Postop Day 2	0.07±0.11 (0.041)	0.05±0.05(0.036)	0.03±0.04 (0.024)	0.06±0.13 (0.023)	0.203
	♦p	0.001**	0.008**	0.022*	0.001**	

♦ : Kruskal Wallis test; ♦ : Wilcoxon Sing Rank test; Mean±SD: Mean±Standard deviation

* p<0.05

** p<0.01

**Fig.1:**
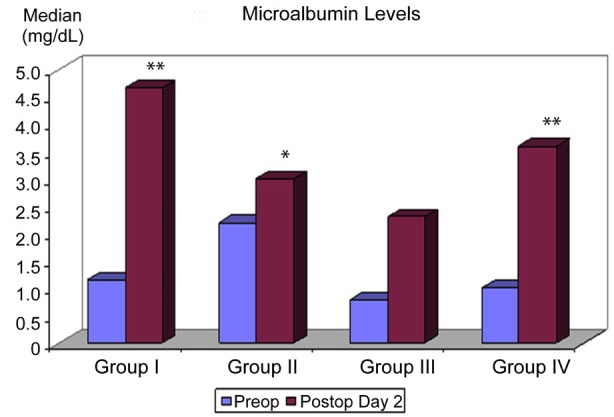
Distribution of urine microalbumin measurements (mg/dl) among groups.

Postoperatively, patients in Group I and IV had a significant increase (p < 0.01) in urinary microalbumin as compared to other groups. Although there was a significant difference within group change in Group II (p < 0.05), between-group increases in Group II and III was not significant (Table-II) (Fig.1).

**Fig.2:**
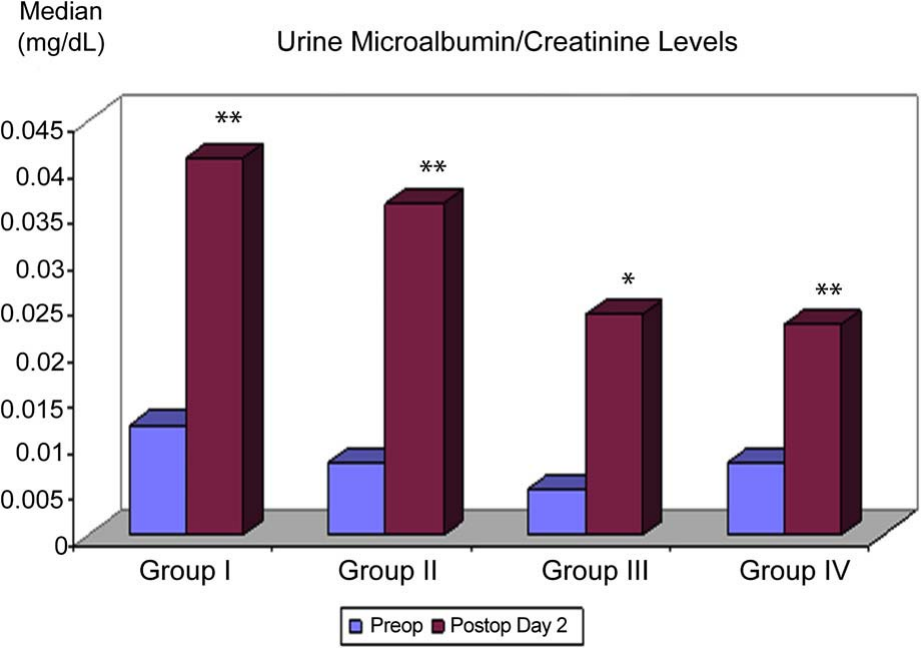
Distribution of urine microalbumin/creatinine ratio measurements (mg/dl) among groups.

A statistically significant increase in urine microalbumin/creatinine ratio postoperatively was observed in Groups I, II, IV (p < 0.01) and III (p<0.05), although no difference existed between the groups in this regard (Table-II) (Fig.2).

**Fig.3:**
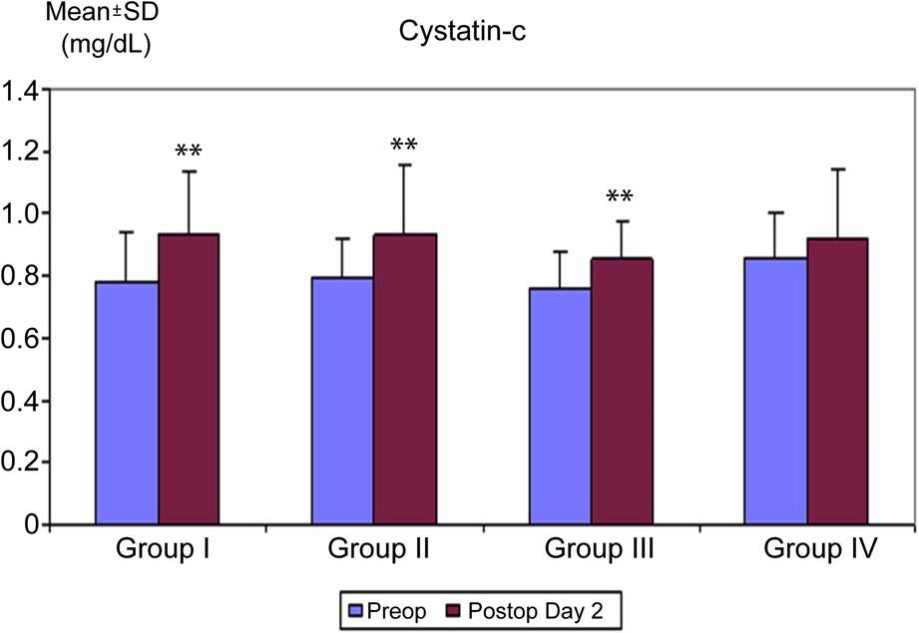
Distribution of cystatin-c measurements (mg/dl) among groups.

There was a significant increase in cystatin-c postoperatively in Groups I, II and III (p < 0.01), with no significant increase in within group comparisons in Group IV (Table-II) (Fig.3). The study groups were similar with respect to the need for additional diuretic use.

## DISCUSSION

The etiology of the kidney dysfunction occurring after open cardiac surgery is multi-factorial and is generally explained on the basis of perioperative low cardiac output or effects of CPB.^5^-^7^

More specific, bypass-related risk factors frequently referred in the literature include cross-clamp time, duration of CPB exceeding 70 minutes in particular^8^, pulsatile vs. non-pulsatile bypass flow^5^, normothermic vs. hyothermic bypass^6^, and bypass surgery with or without pump.^9^ Production of free radicals (superoxide, hydrogen peroxide), hydroxyl radicals, endothelin, free plasma hemoglobin and elastase during CPB may lead to renal injury.^10^,^11^ Non-pulsatile flow, renal hypoperfusion, and hypothermia have been reported to affect renal functions adversely.^12^,^13^

However, several recent studies suggest an increased risk of renal injury due to hemodilution with hematocrit levels below 25%.^14^,^15^ In our sample, the study groups did not exhibit any significant differences in terms of hemodynamic parameters, blood glucose, hemoglobin, hematocrit levels (minimum Hct of 25), and hourly urine output both during surgery and routine follow-up. Several methods have been advised to prevent kidney failure associated with cardiac surgery including the generally accepted measures such as maintaining a good perfusion pressure during bypass, achieving an optimum hemodilution level, and utilizing pulsatile flow.^13^

Controversial literature data exist regarding the preventive effects of dopamine and mannitol in CPB-related kidney injury when tested with different administration schemes, timing, or routes. However, these studies are generally flawed by small sample sizes and differences in parameters used for monitoring renal functions. Therefore, we aimed at comparing the effects of different doses and routes of administration of mannitol and dopamine through the assessment of widely available laboratory parameters.

Within and between-group comparisons showed no significant differences in urine creatinine levels except within Group II (p < 0.039). But, we observed that Group III had the better results than the other groups in according to the other three tests:

There was a significant increase in postoperative microalbumin levels in Group I, IV (p < 0.01) and II (p<0.05). Within group changes in Group I, II, IV (p<0.01) and III (p<0.05) were significant for urine microalbumin/creatinine ratio. But, the changes in group I and IV than the other groups were more significant (p<0.01). However, no significant superiority for one of these parameters over the other has been found in a previous study.^16^

Urine microalbumin/creatinine ratio and cystatin-c have been reported to provide an earlier and better predictive value for the diagnosis of diabetic nephropathy than 24-hour urinary microalbumin level and serving as a criteria used in the pediatric RIFLE classification.^17^-^21^ In our study, despite a significant increase in urine microalbumin/creatinine ratio in Group IV (p < 0.01), the increase in cystatin-c in this group was not statistically significant (p > 0.05), suggesting that this ratio may represent a more sensitive parameter for renal injury.

## CONCLUSIONS

Dopamine infusion (2 µg/kg/min) in conjunction with the administration of mannitol (1 g/kg) during cardiopulmonary bypass was more effective in the prevention of the adverse effects of prolonged cardiopulmonary bypass on renal functions. Although studies involving larger sample populations are warranted, our findings in patients with normal renal function seem to shed some light on the methods to preserve existing renal reserve, particularly in patients with chronic renal failure.
